# High prevalence of diabetes mellitus and impaired glucose tolerance in liver cancer patients: A hospital based study of 4610 patients with benign tumors or specific cancers

**DOI:** 10.12688/f1000research.8457.1

**Published:** 2016-06-16

**Authors:** Chen Roujun, Yi Yanhua, Li Bixun

**Affiliations:** 1 Department of Internal Medicine, Affiliated Tumor Hospital of Guangxi Medical University, Guangxi, China; 2 School for International Education, Guangxi Medical University, Guangxi, China

**Keywords:** Prevalence, Diabetes mellitus, Impaired glucose tolerance, Liver cancer, Risk Factor

## Abstract

**Objective**: The prevalence of diabetes mellitus (DM), impaired glucose tolerance (IGT) and impaired fasting glucose (IFG) were hypothesised to be different among different tumor patients. This study aimed to study the association between the prevalence of DM, IGT and IFG and liver cancer, colorectal cancer, breast cancer, cervical cancer, nasopharyngeal cancer and benign tumor.

**Methods**:  A hospital based retrospective study was conducted on 4610 patients admitted to the Internal Medical Department of the Affiliated Tumor Hospital of Guangxi Medical University, China. Logistic regression was used to examine the association between gender, age group, ethnicity , cancer types or benign tumors and prevalence of DM, IFG, IGT.

**Results**: Among 4610 patients, there were 1000 liver cancer patients, 373 breast cancer patients, 415 nasopharyngeal cancer patients, 230 cervical cancer patients, 405 colorectal cancer patients, and 2187 benign tumor patients. The prevalence of DM and IGT in liver cancer patients was 14.7% and 22.1%, respectively. The prevalence of DM and IGT was 13.8% and 20%, respectively, in colorectal cancer patients, significantly higher than that of benign cancers. After adjusting for gender, age group, and ethnicity, the prevalence of DM and IGT in liver cancers patients was 1.29 times (CI :1.12-1.66) and 1.49 times (CI :1.20-1.86) higher than that of benign tumors, respectively.

**Conclusion:** There was a high prevalence of DM and IGT in liver cancer patients.

## Background

High fat and carbohydrate diets, associated with dietary carcinogenesis, contribute to abnormal glucose tolerance and cancer ^1, 2^. Previous studies suggest that metabolic syndrome is associated with a modestly increased risk of second breast cancer events and breast cancer-specific mortality ^3^. Blood glucose level in colorectal cancer patients has been shown to correlate significantly with local tumor malignancy ^4^. Antidiabetic medication such as metformin treatment could significantly lower the risk of colorectal cancer in type 2 diabetes mellitus (T2DM) patients ^5^. However, little is known on the correlation between prevalence of impaired glucose tolerance (IGT), diabetes mellitus (DM) and other types of cancers in comparison with benign tumors.

As an ethnic area, Guangxi Zhuang automonous region (abbreviated as Guangxi) located in southwest China, is well known for its high incidence of liver cancer and nasopharyngeal cancer ^6, 7^. Established risk factors including heavy alcohol consumption, chronic infection with the hepatitis B virus (HBV) or the hepatitis C virus (HCV), tobacco smoking, intake of aflatoxin-contaminated foods for the development of hepatocellular carcinoma (HCC) have been studied in Guangxi over the past several decades ^8^. According to an American study, abnormal glucose tolerance was an independent predictor for cancer mortality ^9^. Therefore, it is essential to investigate the prevalence of DM, IGT, and IFG (impaired fasting glucose) among patients with common types of cancer in such a high incidence area.

This study aimed to investigate the association between the prevalence of DM, IGT and IFG and liver cancer, colorectal cancer, breast cancer, cervical cancer, nasopharyngeal cancer and benign tumors.

## Method

### Data collection

This hospital based study was approved by the Medical Ethics Committee of the Affiliated Tumor Hospital of Guangxi Medical University, China. The data were retrieved from the Internal Medical Department of the hospital. All personal identification was encrypted.

Patients hospitalized in the Internal Medical Department between 2010 and 2012 with complete records of fasting plasma glucose (FPG) and 2-h postprandial glucose (2hPPG) upon hospitalization were included in this study. Clinical data were collected, including gender, age, ethnicity, DM history, diagnosis, pathological diagnosis, FPG, and 2hPPG. All diagnoses of cancers and benign tumors were based on CT scan results, endoscopic biopsy or surgical resection. Those who had serious cardiocelebral diseases, liver or kidney dysfunction and other conditions that may influence diabetic or prediabetic state were excluded from this study.

### Measurement of glucose level

The glucose levels were included in the retrospective data. The 1999 World Health Organization (WHO) diagnostic criteria were used to diagnose DM, IFG and IGT ^10^. Results of plasma glucose testing were categorized as follows. Normal glucose tolerance (NGT): FPG <6.1 mmol/L, and 2hPPG <7.8 mmol/L; Diabetes mellitus (DM): FPG ≥7 .0 mmol/L, 2hPPG ≥11.1 mmol/L, and with DM medical history. Impaired Fasting blood glucose (IFG): FPG ≥6.1 mmol/L and <7.0 mmol/L with the exclusion of cases with DM history or 2hPPG ≥11.1 mmol/L. Impaired glucose tolerance (IGT): FPG<7.0 mmol/L, and 2hPPG ≥7.8 mmol/L and <11.1 mmol/L with the exclusion of cases with DM history.

### Statistical analysis

Descriptive data were presented as frequencies and percentages, followed by a chi-square test or Fisher’s test as appropriate. Logistic regression was used to examine the association between gender, age group, ethnicity and cancer types or benign tumors and prevalence of DM, IFG, IGT. Data analyses were performed using the R language and environment (version 3.1.0) ^11^ and the Epicalc package (version 3.1.1.2) ^12^.

## Results

Patient data Click here for additional data file. Copyright: © 2016 Roujun C et al. 2016 Data associated with the article are available under the terms of the Creative Commons Zero "No rights reserved" data waiver (CC0 1.0 Public domain dedication).

Data analysis using R Click here for additional data file. Copyright: © 2016 Roujun C et al. 2016 Data associated with the article are available under the terms of the Creative Commons Zero "No rights reserved" data waiver (CC0 1.0 Public domain dedication).

**1. Demographic description and prevalence of DM, IGT, and IFG among different cancers and benign tumors:**

**Figure 1.**
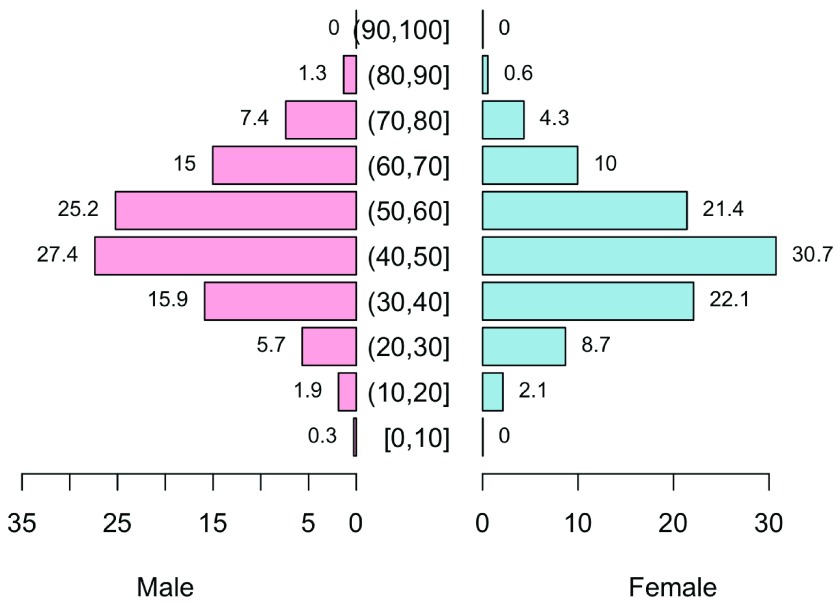
Population pyramid distribution by gender and age group.

A total of 4610 patients were included in this study. Figure 1 and Table 1 summarize the demographic characteristics of the patients, among whom 1000 were liver cancer patients, 373 breast cancer patients, 415 nasopharyngeal cancer patients, 230 cervical cancer patients, 405 colorectal cancer patients, and 2187 benign tumor patients. The patients in the 40–60 year age group comprised more than 50% of the total patients studied. Liver cancer, nasopharyngeal cancer, and colorectal cancer patients were predominantly male. Age and ethnic distribution among the studied groups were statistically different (P<0.001).

**Table 1.** Demographic information and prevalence of DM, IFG, and IGT among patients with specific cancers or benign tumors.

	Liver cancer (N=1000)	Breast cancer (N=373)	Nasopharyngeal cancer (N=415)	Cervical cancer (N=230)	Colorectal cancer (N=405)	Benign tumors (N=2187)
Gender***						
Male	878 (87.8)	0(0.0)	294 (70.8)	0(0.0)	255 (63.0)	834 (38.1)
Female	122 (12.2)	373(100.0)	121 (29.2)	230(100.0)	150 (37.0)	1353 (61.9)
Age (median,IQR)***	49 (42,58)	48 (40,56)	46 (39,54)	48 (42,55)	59 (48,68)	46 (36,57)
Ethnic groups***					
Han	607 (60.7)	256 (68.6)	289 (69.6)	153 (66.5)	305 (75.3)	1438 (66.8)
other ethnicity	20 (2.0)	12 (3.2)	6 (1.4)	9 (3.9)	10 (2.5)	66 (3.1)
Zhuang	373 (37.3)	105 (28.2)	120 (28.9)	68 (29.6)	90 (22.2)	650 (30.2)
DM***	147 (14.7)	39 (10.5)	21 (5.1)	18 (7.8)	56 (13.8)	197 (9.1)
IFG**	28 (2.8)	27 (7.2)	25 (6.0)	6 (2.6)	22 (5.4)	98 (4.5)
IGT***	221 (22.1)	48 (12.9)	53 (12.8)	28 (12.2)	81 (20.0)	262 (12.0)

Data were given as n (%). * p-value<0.05; ** p-value<0.01; *** p-value < 0.001.

The prevalence of DM and IGT in liver cancer patients was 14.7% and 22.1%, respectively; 13.8% and 20% in colorectal cancer patients, which were significantly higher than that of benign cancers.

**2. Association between prevalence of DM and cancer types and benign tumors**

As shown in Table 2, adjusted for gender, ethnicity, and age group, the prevalence of DM in liver cancers patients was 1.29 times higher than that of benign tumors; male patients were 1.49 times of higher than that of female patients. Using the 0–30 year as reference, the prevalence of DM increased with age. The peak risk ratio was 10.3 times higher in the 61–70 year age group than that of 0–30 year age group. The Han ethnic group was 1.38 times more likely to have DM in comparison with the Zhuang ethnic group.

**Table 2.** Association between prevalence of DM and specific cancers or benign tumors.

	crude OR(95%CI)	adj. OR(95%CI)	P(LR-test)
Diagnosis: ref.=benign tumor			< 0.001
Breast cancer	1.17 (0.81,1.68)	1.28 (0.87,1.89)	
Cervical tumor	0.85 (0.51,1.4)	0.88 (0.52,1.48)	
Colorector cancer	1.6 (1.17,2.2)	0.95 (0.69,1.33)	
Liver cancer	1.72 (1.37,2.16)	1.29 (1.12,1.66)	
Nasopharyngeal cancer	0.53 (0.34,0.85)	0.46 (0.29,0.74)	
Gender: male vs female	1.74 (1.44,2.12)	1.49 (1.17,1.89)	0.001
Ethnicity: ref.=Zhuang			0.018
Han	1.47 (1.18,1.83)	1.38 (1.1,1.74)	
Other ethnicity	1.34 (0.73,2.45)	1.36 (0.73,2.53)	
Age group: ref.=(0,30)			< 0.001
(31,40)	1.88 (0.9,3.97)	1.87 (0.89,3.95)	
(41,50)	3.9 (1.96,7.79)	3.75 (1.87,7.52)	
(51,60)	8.94 (4.53,17.66)	8.14 (4.1,16.17)	
(61,70)	11.36 (5.69,22.69)	10.3 (5.13,20.68)	
>70	8.46 (4.09,17.54)	7.28 (3.49,15.18)	

**3. Association between prevalence of IGT and cancer types and benign tumors**

As shown in Table 3, adjusted for gender, ethnicity, and age group, the prevalence of IGT in liver cancer patients was 1.49 times higher than that of benign tumors; male patients were 1.71 times higher than that of female patients. Using the 0–30 year as reference, there were an obvious age-dependent relationship between age groups and prevalence of IGT. There were no significant difference among the ethnic groups in terms of IGT prevalence (P=0.421).

**Table 3.** Association between prevalence of IGT and specific cancers or benign tumors.

	crude OR(95%CI)	adj. OR(95%CI)	P(LR-test)
Diagnosis: ref.=benign tumors			0.002
Breast cancer	1.07 (0.77,1.48)	1.28 (0.9,1.82)
Cervical tumor	1 (0.66,1.52)	1.17 (0.76,1.8)
Colorector cancer	1.81 (1.37,2.38)	1.25 (0.94,1.67)
Liver cancer	2.05 (1.68,2.5)	1.49 (1.2,1.86)
Nasopharyngeal cancer	1.06 (0.77,1.45)	0.88 (0.64,1.22)	
Gender: male vs female	2.01 (1.7,2.38)	1.71 (1.39,2.1)	< 0.001
Ethnicity: ref.=Zhuang			0.421
Han	1.07 (0.9,1.28)	1.04 (0.86,1.24)
Other ethnicity	0.69 (0.38,1.25)	0.71 (0.39,1.3)
Age group: ref.=(0,30)			< 0.001
(31,40)	1.66 (1.06,2.6)	1.55 (0.98,2.43)	
(41,50)	2.63 (1.73,3.99)	2.34 (1.53,3.58)	
(51,60)	3.18 (2.09,4.84)	2.67 (1.74,4.09)	
(61,70)	3.83 (2.47,5.94)	3.19 (2.04,4.98)	
>70	4.62 (2.89,7.38)	3.79 (2.35,6.12)	

**4. Association between prevalence of IFG and cancer types and benign tumors**

As shown in Table 4, adjusted for gender, ethnicity, and age group, the prevalence of IFG in liver cancers patients was 0.55 times lower than that of benign tumors. Using the 0–30 year as reference, the prevalence of IFG increased with age. The Han ethnic group was 1.59 times higher in prevalence of IFG in comparison with the Zhuang ethnic group.

**Table 4.** Association between prevalence of IFG and specific cancers or benign tumors.

	crude OR(95%CI)	adj. OR(95%CI)	P(LR-test)
Diagnosis: ref.=benign tumor			0.003
Breast cancer	1.64 (1.05,2.54)	1.57 (0.99,2.51)	
Cervical tumor	0.56 (0.24,1.3)	0.53 (0.23,1.24)	
Colorector cancer	1.21 (0.75,1.94)	0.92 (0.57,1.51)	
Liver cancer	0.6 (0.39,0.93)	0.55 (0.35,0.87)	
Nasopharyngeal cancer	1.34 (0.86,2.11)	1.26 (0.79,2.02)
Gender: Male vs Female	0.96 (0.73,1.27)	1.08 (0.77,1.52)	0.641
Ethnicity: ref.=Zhuang			0.023
Han	1.73 (1.23,2.44)	1.59 (1.13,2.26)
Other ethnicity	1.34 (0.52,3.46)	1.25 (0.48,3.24)
Age group: ref.=(0,30)			< 0.001
(31,40)	1.99 (0.86,4.59)	2.06 (0.89,4.78)	
(41,50)	3.19 (1.45,7)	3.35 (1.52,7.41)	
(51,60)	2.97 (1.34,6.61)	3.13 (1.4,7.03)	
(61,70)	3.34 (1.45,7.67)	3.46 (1.49,8.01)	
>70	5.01 (2.13,11.78)	4.89 (2.06,11.65)	

## Discussion

This study showed that the prevalence of DM and IGT in liver cancer patients was 14.7% and 22.1%, respectively. After adjustment for gender, age group, and ethnicity, the prevalence of DM and IGT in liver cancer patients was 1.29 times (CI :1.12–1.66) and 1.49 times (CI :1.20–1.86) higher, respectively, than that of benign tumors. In comparison, the prevalence of DM and IGT in patients with breast cancer, cervical cancer, colorectal cancer, and nasopharyngeal cancer was not significantly different from that of benign tumors after adjusted for gender, age group, and ethnicity.

A previous study suggested high blood glucose levels in colorectal cancer patients with larger tumor diameters and lower tumor differentiation ^4^. Our study also found that the prevalence of DM and IGT was high in colorectal cancer patients in the preliminary analysis, although no statistical difference was found after adjustments for other factors. So far, there have been very few studies on the relationship between prevalence of DM and IGT and liver cancer. However, the relationship between obesity, overweight and higher rates of death due to cancer of esophagus, colon, rectum, liver, gallbladder, pancreas and kidney, non-Hodgkin’s lymphoma, and multiple myeloma has been well established by a large sample prospective study ^13^. The reason why a high prevalence of DM and IGT is correlated with liver cancer may be explained as follows: As a regulator of energy storage and metabolism, insulin was produced and secreted by pancreatic *β* cells, which stimulated glucose uptake by adipose tissue and muscle, suppressing the release of glucose from the liver. Desensitization of tissues to insulin and insulin resistance led to a compensating increase in pancreatic insulin production called hyperinsulinemia, which may have possible direct oncogenic effects on proliferative and anti-apoptosis signaling in cancer cells.

High prevalence of DM and IGT correlated with liver cancer had important implications both in prevention and treatment of liver cancer. Metformin, a commonly used drug for type 2 diabetes, led to a lower incidence and mortality of breast cancer ^14– 18^. In a similar way, the control of high blood glucose levels would possibly improve clinical outcomes of liver cancer patients.

### Strengths and limitations

As a hospital based study recently conducted in Guangxi with a high incidence of liver cancers, this study provided evidence for government and public health planners which may be useful in the prevention of liver cancer. Further prospective cohort studies should be conducted to learn the strength of association between DM, IGT and liver cancer; moreover, to learn how much the control of diabetic or pre-diabetic conditions contributes to the prevention and treatment of liver cancer.

## Conclusion

This study showed a high prevalence of DM and IGT in liver cancer patients. The control of diabetic or pre-diabetic conditions may contribute to the prevention and treatment of liver cancer.

## Data and software availability

*F1000Research*: Dataset 1. Patient data, 10.5256/f1000research.8457.d122894 ^19^

*F1000Research*: Dataset 2. Data analysis using R, 10.5256/f1000research.8457.d122895 ^20^
