# Supplementary material for: High prevalence of diabetes mellitus and impaired glucose tolerance in liver cancer patients: A hospital based study of 4610 patients with benign tumors or specific cancers
Source: F1000Res. 2016 Jun 16;5:1397. [Version 1] doi: 10.12688/f1000research.8457.1 (PMC4995685; doi:10.12688/f1000research.8457.1)
Supplement: Peer review history

# Peer review: Referee response for version 1

**DOI:** 10.5256/f1000research.9106.r14411

**Published:** 2016-08-23

**Authors:** Nirun Intarut

**Affiliations:** 1 Clinical Epidemiology Unit, Faculty of Medicine, Mahasarakham University, Maha Sarakham, Thailand

This is a retrospective study that was carried out in a hospital in China, involving 4610 participants. In summary, this study investigated the association between the DM, IGT and IFG and liver cancer, colorectal cancer, breast cancer, cervical cancer, nasopharyngeal cancer and benign tumors. The study shows the high prevalence of DM and IGT in liver cancer.

 My suggestion if possible, is to include tobacco consumption or exposure to secondhand smoke into this study. Because there is a high prevalence of smoking and also high exposure to secondhand smoke in China ^1-3^, there could be evidence to show its association between DM and liver cancer ^4-7^.

I have read this submission. I believe that I have an appropriate level of expertise to confirm that it is of an acceptable scientific standard.

## References

[1] NanYXiZYangYWangLLTuMWWangJJJiangY: [The 2015 China Adult Tobacco Survey: exposure to second-hand smoke among adults aged 15 and above and their support to policy on banning smoking in public places]. *Zhonghua Liu Xing Bing Xue Za Zhi*.2016;37(6) :810-5 2734610710.3760/cma.j.issn.0254-6450.2016.06.014

[2] ZengJYangSWuLWangJWangYLiuMZhangDJiangBHeY: Prevalence of passive smoking in the community population aged 15 years and older in China: a systematic review and meta-analysis. *BMJ Open*.2016;6(4) : 10.1136/bmjopen-2015-009847 e009847 10.1136/bmjopen-2015-009847 27059465PMC4838695

[3] YangTYuLJiangSFengXXianHCottrellRRRockettIR: Household smoking restrictions among urban residents in China: individual and regional influences. *Int J Public Health*.2015;60(4) : 10.1007/s00038-015-0672-0 479-86 10.1007/s00038-015-0672-0 25838120

[4] PangQQuKZhangJXuXLiuSSongSWangRZhangLWangZLiuC: Cigarette smoking increases the risk of mortality from liver cancer: A clinical-based cohort and meta-analysis. *J Gastroenterol Hepatol*.2015;30(10) : 10.1111/jgh.12990 1450-60 10.1111/jgh.12990 25967392

[5] MeyerJRohrmannSBoppMFaehD: Impact of Smoking and Excess Body Weight on Overall and Site-Specific Cancer Mortality Risk. *Cancer Epidemiol Biomarkers Prev*.2015;24(10) : 10.1158/1055-9965.EPI-15-0415 1516-22 10.1158/1055-9965.EPI-15-0415 26215293

[6] ParkSJeeSHShinHRParkEHShinAJungKWHwangSSChaESYunYHParkSKBoniolMBoffettaP: Attributable fraction of tobacco smoking on cancer using population-based nationwide cancer incidence and mortality data in Korea. *BMC Cancer*.2014;14: 10.1186/1471-2407-14-406 406 10.1186/1471-2407-14-406 24902960PMC4090397

[7] BlakelyTBarendregtJJFosterRHHillSAtkinsonJSarfatiDEdwardsR: The association of active smoking with multiple cancers: national census-cancer registry cohorts with quantitative bias analysis. *Cancer Causes Control*.2013;24(6) : 10.1007/s10552-013-0204-2 1243-55 10.1007/s10552-013-0204-2 23580085

# Peer review: Referee response for version 1

**DOI:** 10.5256/f1000research.9106.r14413

**Published:** 2016-06-24

**Authors:** He Jianhui

**Affiliations:** 1 Department of Health Economics and Health Management, Kunming Medical University, Kunming, China

The article entitled “High prevalence of diabetes mellitus and impaired glucose tolerance in liver cancer patients: A hospital based study of 4610 patients with benign tumors or specific cancers” is very meaningful on providing evidence for government to prevent liver cancer in China. They found that the prevalence of DM and IGT in liver cancers patients was significantly higher than in benign tumors. Overall, I appreciate the study. The following is some comments and suggestions on the study topic.

 Some studies ^1^^-^^3^ showed that DM is an independent risk factor of hepatocellular carcinoma, but some other studies ^4^^,^^5^ provided an inconsistent conclusion: only among people who were infected with both HBV and HCV or alcoholic hepatitis can this increase the risk of hepatocellular carcinoma. Furthermore, a systematic review ^6^ pointed out that among 23 case control or follow up studies, only less than 40% of studies indicated that DM is an independent risk factor of hepatocellular carcinoma, and also few studies can exclude the influence of food habits and obesity etc. The reason for this inconsistency is not clear, and needs further study. DM possibly is not an independent risk factor on hepatocellular carcinoma, but among people who were hepatitis virus infected or alcoholic with DM will increase the probability getting hepatocellular carcinoma.

 According to the above, I suggest that, if available, please add the information about some habits such as alcoholic beverage drinking history, some indices including BMI for example to exclude the influence of such factors which are both related to DM and hepatocellular carcinoma, and the status of virus hepatitis in the analysis. Moreover, a retrospective study cannot provide the causal relationship between DM and hepatocellular carcinoma, if we want to know the relationship clearly, a population based case control and prospective cohort study are needed.

I have read this submission. I believe that I have an appropriate level of expertise to confirm that it is of an acceptable scientific standard.

## References

[1] LaiMSHsiehMSChiuYHChenTH: Type 2 diabetes and hepatocellular carcinoma: A cohort study in high prevalence area of hepatitis virus infection. *Hepatology*.2006;43(6) : 10.1002/hep.21208 1295-302 10.1002/hep.21208 16729295

[2] ChenCLYangHIYangWSLiuCJChenPJYouSLWangLYSunCALuSNChenDSChenCJ: Metabolic factors and risk of hepatocellular carcinoma by chronic hepatitis B/C infection: a follow-up study in Taiwan. *Gastroenterology*.2008;135(1) : 10.1053/j.gastro.2008.03.073 111-21 10.1053/j.gastro.2008.03.073 18505690

[3] DavilaJAMorganROShaibYMcGlynnKAEl-SeragHB: Diabetes increases the risk of hepatocellular carcinoma in the United States: a population based case control study. *Gut*.2005;54(4) : 10.1136/gut.2004.052167 533-9 10.1136/gut.2004.052167 15753540PMC1774454

[4] FujinoYMizoueTTokuiNYoshimuraT: Prospective study of diabetes mellitus and liver cancer in Japan. *Diabetes Metab Res Rev*.17(5) :374-9 1174714210.1002/dmrr.214

[5] El-SeragHBRichardsonPAEverhartJE: The role of diabetes in hepatocellular carcinoma: a case-control study among United States Veterans. *Am J Gastroenterol*.2001;96(8) : 10.1111/j.1572-0241.2001.04054.x 2462-7 10.1111/j.1572-0241.2001.04054.x 11513191

[6] El-SeragHBHampelHJavadiF: The association between diabetes and hepatocellular carcinoma: a systematic review of epidemiologic evidence. *Clin Gastroenterol Hepatol*.2006;4(3) : 10.1016/j.cgh.2005.12.007 369-80 10.1016/j.cgh.2005.12.007 16527702
